# Association between urinary heavy metal mixtures and overactive bladder risk in the U.S. adult population: a cross-sectional study

**DOI:** 10.3389/fpubh.2025.1577413

**Published:** 2025-11-13

**Authors:** Zhenzhen Liu, Zikai Guo, Husong Su, Sheng Xue, Bing Xiong

**Affiliations:** 1 Department of Obstetrics and Gynecology, Affiliated Hospital of Zunyi Medical University, Zunyi, China; 2 School of Medicine, Bengbu Medical University, Bengbu, China; 3 School of Anesthesiology, Zunyi Medical University, Zunyi, China; 4 Department of Urology, First Affiliated Hospital of Bengbu Medical University, Bengbu, China

**Keywords:** heavy metal, OAB, WQS model, BKMR model, RCS

## Abstract

The existing body of literature consistently underscores the adverse health implications associated with exposure to toxic metals among humans. There is still a substantial knowledge gap about how concurrent exposure to metal combinations affects the prevalence of overactive bladder (OAB). Our detailed examination focused on the complex associations between simultaneous heavy metal exposure and OAB in adults. By utilizing data from 7,719 adults in the National Health and Nutrition Examination Survey (NHANES), we assessed both individual and combined effects. From all participants, 1,574 (20.39%) were diagnosed with OAB. The research found that elevated urinary levels of cadmium, cobalt, lead, and antimony were associated with a higher risk of OAB. Conversely, urinary barium exhibited a noteworthy protective effect against OAB. Additionally, the weighted quantile sum regression and BKMR models consistently underscored the positive influence of being exposed to multiple urinary metals at the same time on the risk of OAB. The impact was more evident among young and middle-aged people and those who have consumed alcohol, as opposed to older adults and abstainers. The BKMR analysis additionally unveiled potential interactions among specific components of metal mixtures contributing to OAB. The g computation model based on quantiles verified the harmful impacts of metal combinations on OAB. Furthermore, the risk of OAB was positively correlated with Cd and Co, as revealed by restricted cubic spline regression. The research reveals that being exposed to various heavy metals simultaneously significantly boosts the risk of OAB. To thoroughly validate these results, prospective studies are essential due to the limitations of the NHANES study design.

## Graphical abstract

**Figure:**
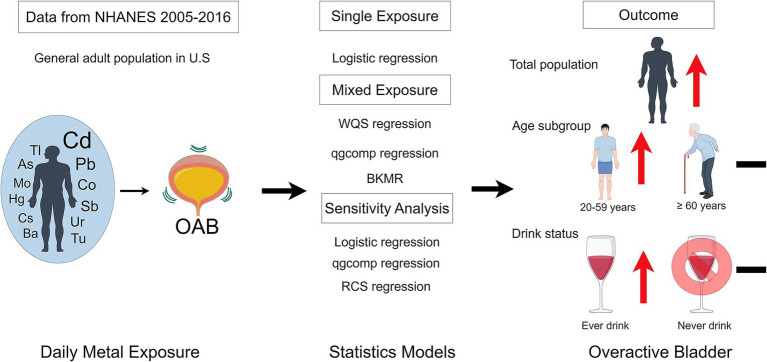

## 1 Introduction

Overactive bladder (OAB) is characterized by symptoms like frequent and urgent urination, nighttime urination, and possibly urgent urinary incontinence. Epidemiological studies indicate that 12–17% of adults residing in the community experience these symptoms, with an increasing prevalence among both sexes with aging (1–3). Prevalence rates have been documented to reach 30–40% in other studies (4). OAB symptoms adversely affect patients’ daily functioning and quality of life (5, 6). In economic terms, it results in lower productivity and higher healthcare costs (7). In spite of recent progress, the precise cause of OAB is still unknown, and none of the available treatments offer a conclusive cure.

The environment is widely contaminated with heavy metals, which are present in drinking water, soil, air, dust, the food chain, and industrial products (8, 9). Exposure to metals in the environment is a significant global public health issue because it is associated with numerous urinary system diseases, such as urogenital cancer (10, 11), kidney health (12), and kidney stones (13). Furthermore, molecular damage induced by metals affects precise neuronal functions by interacting with synaptic vesicles, ion channels, neurotransmitter metabolism, and intracellular signals (14–16). Furthermore, it might suppress the expression of numerous genes required for the differentiation and functioning of sensory neurons. Recent research indicates that dysfunction in supraspinal regions responsible for sensory processing and efferent control might lead to OAB (17). Given the crucial function of the brain in managing LUT (18), metals can alter estrogen and androgen receptor levels and disrupt sex hormone balances (19–21). Although hormonal shifts and nervous system damage are known factors in OAB, the connection between metal exposure and OAB remains uncertain.

Additionally, the importance of heavy metals in the development of bladder function is being increasingly acknowledged. The study indicated that exposure to increased cadmium (Cd) and lead (Pb) levels is related to urinary incontinence in women (22). However, this study focused on single metal effects, potentially underestimating or overestimating the effect of individual metals. Because heavy metals are found in the environment together and may interact in ways that are antagonistic, additive and synergistic, focusing solely on individual metals may not adequately reflect disease onset and progression. Consequently, investigating the joint influence of metals on OAB is necessary.

## 2 Materials and methods

### 2.1 Data collection and study population

**Figure 1:**
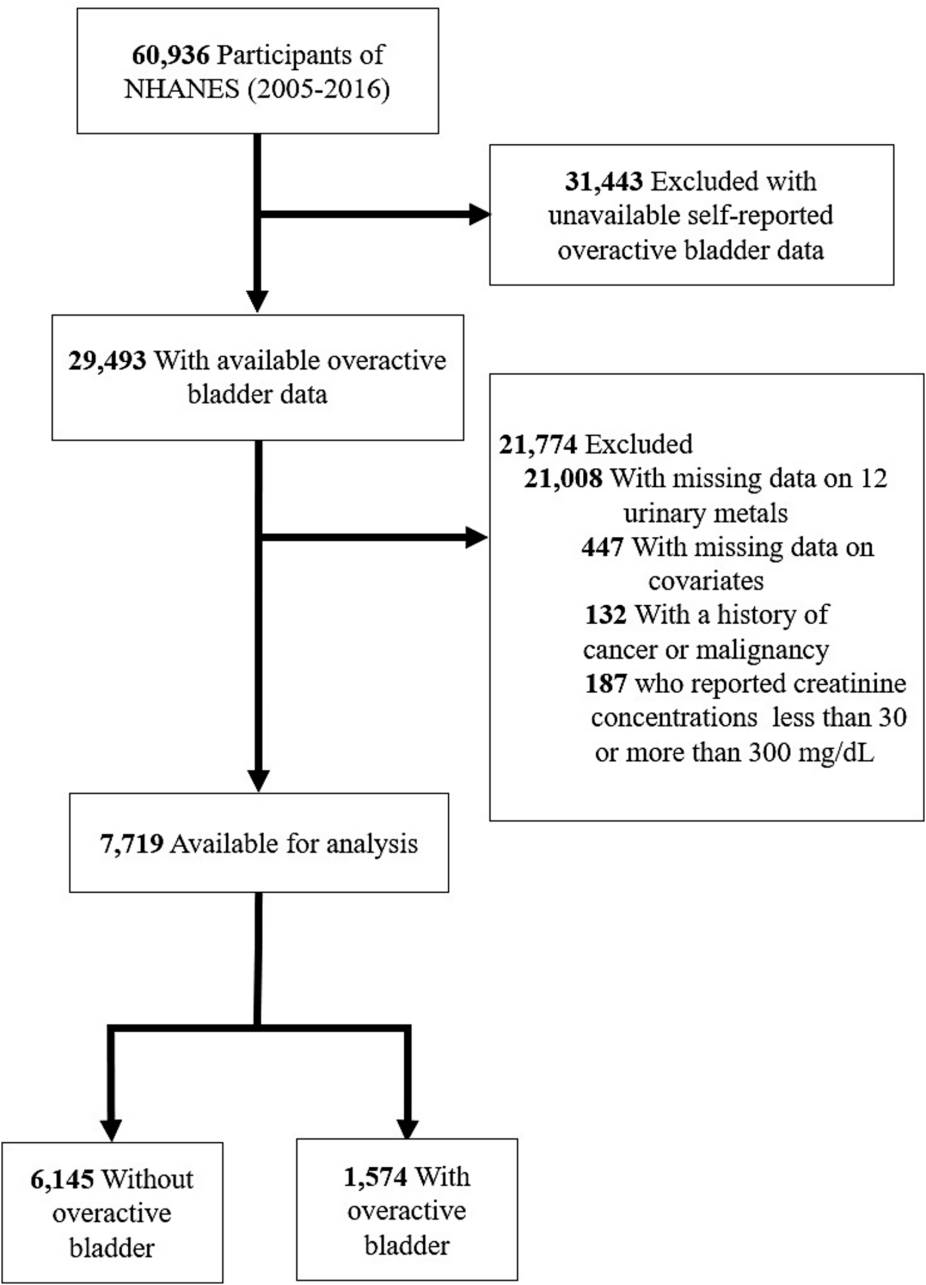
Flow diagram of the screening and enrollment of study participants.

Through a stratified, multistage probability design, The National Health and Nutrition Examination Survey (NHANES) conducts a detailed cross-sectional survey to assess the health and nutrition of the noninstitutionalized population in the United States (23). All participants gave informed consent in accordance with NHANES protocols, approved by its institutional review board (24). Data for this study were extracted from six NHANES cycles (2005–2006, 2007–2008, 2009–2010, 2011–2012, 2013–2014, and 2015–2016). In this analysis, individuals who were unsure of their OAB status, lacked information on 12 metals or covariates, or were below the age of 20 years were excluded. Among the 7,719 participants, 1,574 were found to have OAB (Figure 1).

### 2.2 Criteria for OAB

OAB, as described by the International Continence Society, involves urinary urgency, often with increased frequency and nocturia, and can include urgency urinary incontinence (UUI), as long as there is no urinary tract infection or other obvious pathology (25). Trained researchers collected this information through face-to-face interviews based on structured questionnaires. Participants were queried about the urgency of urinary incontinence (UUI) by asking if they had experienced any involuntary urine leakage or loss in the past year due to an urgent need to urinate, preventing them from reaching a toilet in time. The intensity of UUI was assessed by asking about how often these incidents happened. Inquiries were used to evaluate the severity of nocturia by asking “How many times did you usually wake up to urinate over the past 30 days?” The assessment of OAB was done using the OABSS, where a score of 3 or higher indicated the presence of OAB (26). For more detailed information, refer to Supplementary Figure S1.

### 2.3 Urinary concentration of metals

Using metal-free containers, 29 spot urine samples were obtained at mobile examination centers. Frozen at −20°C, the specimens were forwarded to the Division of Environmental Health Laboratory Sciences, NCEH, for further examination. Using inductively coupled plasma dynamic reaction cell mass spectrometry, the urinary levels of 15 elements were measured, including arsenic (As), antimony (Sb), mercury (Hg), barium (Ba), cobalt (Co), cesium (Cs), lead (Pb), molybdenum (Mo), thallium (Tl), tungsten (Tu), and uranium (Ur). Element concentrations were determined in micrograms per liter. To achieve multi-internal standardization, urine samples were diluted 1:9 with a 2% (v/v) solution of double-distilled concentrated nitric acid that included iridium and rhodium (27). Our study estimated metal concentrations in urine that were below the detection limit by using the LOD divided by the square root of two. Also, the levels of metals in urine were adjusted based on creatinine and reported as micrograms per gram of creatinine. Consult the NHANES Lab Protocol web page for in-depth experimental methods and protocols.

### 2.4 Covariates

Consistent with previous studies (28–30), covariates encompass interview age (20–59 years, 60 years and above), sex (male, female), race/ethnicity (Mexican American, Other Hispanic, non-Hispanic), and education level (high school or lower, higher than high school), family poverty-income ratio (≤1, >1), marital status (married/living with partner, widowed/divorced/separated, never married), body mass index (<25, ≥25 kg/m2), alcohol use, smoking status, hypertension, diabetes, stroke, urine creatinine, and NHANES cycles.

### 2.5 Statistical analysis

Baseline comparisons according to OAB status employed t-tests for continuous data and chi-square tests for categorical data. For continuous variables, mean ± standard deviation (SD) is reported, and for categorical variables, n (%). To adjust the right-skewed distribution of heavy metal content in the human body, the urinary metal data underwent an inverse transformation to approximate a normal distribution for continuous variables and were then split into four quartiles: Q1, Q2, Q3, and Q4.

To evaluate the relationships, Pearson correlation analysis was applied to inverse-transformed urinary metal concentrations. Pearson’s correlation coefficients were grouped into strong (*r* > 0.8), medium (0.3 < *r* ≤ 0.8), or weak (*r* ≤ 0.3). A stratified analysis was conducted by age and alcohol consumption, categorizing participants into young and middle-aged (20 ≤ Age < 60) and older adult (Age ≥ 60) groups, as well as ever-drinkers and never-drinkers groups.

The initial step involved using a multivariable logistic regression analysis to study the effect of individual metals on OAB risk. Outcomes were reported as odds ratios (ORs) with 95% confidence intervals (CIs), using the first quartile (Q1) as the reference group. Adjustments were made to the models, including age, sex, race/ethnicity, education levels, poverty-income ratio, marital status, body mass index, alcohol consumption, smoking habit, hypertension, diabetes, stroke, urinary creatine concentrations, and NHANES cycles.

The overall influence of mixed metals on the outcome was assessed using Weighted Quantile Sum (WQS) regression analysis (31), effectively capturing the characteristics of environmental mixtures. A 40:60 ratio was used to randomly divide the dataset into a training set and a validation set. After determining the weights of individual metals with 1,000 bootstrap iterations on the training dataset, the mixture’s significance was assessed using the validation dataset. The WQS index, incorporating weighted sums of concentrations for each metal by using the R package “gWQS.” The WQS index, which varies from 0 to 1, evaluated the combined exposure level of 12 urinary metals, with significant weights indicating their relative importance. The results assessed the effect of introducing one quantile of mixed metals on the risk associated with OAB.

Selected for its robustness and rising relevance in statistical analysis, the Bayesian kernel machine regression (BKMR) model is utilized (32, 33), This method precisely estimates the multivariable exposure-response function, accounting for nonlinearity, non-additivity, and confounding variables, including bias-inducing factors. The study analyzed the effect of metal mixtures on OAB by evaluating changes in OAB estimates linked to each 5-percentile adjustment in the median concentration of these mixtures. The posterior inclusion probability, with a threshold of 0.1, assessed each metal mixture component’s relative contribution to the outcome. Using the R package ‘bkmr,’ the BKMR model estimate was achieved through 20,000 iterations.

To ensure result reliability, multiple sensitivity analyses were conducted. Initially, each logistic regression model included all urinary metals separately to consider the possible effects of other metals. Additionally, the approach of WQS, which evaluates exposures related to the outcome consistently, was applied. Our proposal involved employing the quantile-based g computation (qgcomp) model, by using R package “qgcomp” (34). To overcome this limitation. Without assuming directional homogeneity, this approach allows for the identification of both positive and negative weights for each factor in the mixtures. In order to more effectively understand the relationship between metal exposure levels and OAB, ‘rms’ package in R was used to carry out a restricted cubic spline regression analysis (35). This method evaluates the linear association between metals and OAB risk, in addition to capturing the non-linear dynamics between these variables. Using the Akaike information criterion, the number of knots was selected to optimize model fit and minimize overfitting (36), which resulted in selecting three knots at the 10th, 50th, and 90th percentiles. Data analysis was conducted using R software (version 4.3.1; Lucent Technologies Inc.). A *p*-value below 0.05 indicated statistical significance.

## 3 Results

### 3.1 Participant characteristics

7,719 adults from six NHANES cycles, 1,574 (20.39%) were diagnosed with OAB, while the others were not. Table 1 shows the initial characteristics of participants, differentiating between those with OAB and those without. The groups exhibited significant differences in age, sex, race/ethnicity, education, Poverty Income Ratio, marital status, body mass index, smoking habits, alcohol consumption, diabetes, stroke, hypertension, and urine creatinine distributions.

**Table 1:** Characteristics of Participants in the NHANES 2005–2016 cycles.

Variable	Participants			*p* value
	Total (*n* = 7,719)	Non-OAB (*n* = 6,145)	OAB (*n* = 1,574)	
Age, *N* (%)				
20 ~ 59	1870 (24.2%)	1,192 (19.4%)	781 (49.6%)	< 0.0001
≥60	5,849 (75.8%)	4,953 (80.6%)	793 (50.4%)	
Sex, *N* (%)				< 0.0001
Female	3,697 (48.9%)	2,818 (46.6%)	879 (61.6%)	
Male	4,022 (51.1%)	3,327 (53.4%)	695 (38.4%)	
BMI (kg/m^2^), *N* (%)				< 0.0001
18 ~ 23	5,934 (76.9%)	4,633 (75.4%)	1,328 (84.4%)	
≥24	1785 (23.1%)	1,512 (24.6%)	246 (15.6%)	
Race/ethnicity, *N* (%)				< 0.0001
Mexican American	633 (8.2%)	516 (8.4%)	116 (7.4%)	
Non-Hispanic Black	841 (10.9%)	602 (9.8%)	263 (16.7%)	
Non-Hispanic White	5,396 (69.9%)	4,326 (70.4%)	1,041 (66.1%)	
Other Hispanic	394 (5.1%)	314 (5.1%)	77 (4.9%)	
Other Race/Multiracial	455 (6.1%)	387 (6.3%)	787 (5.0%)	
PIR, *N* (%)				0.28
> 1	6,646 (86.1%)	5,334 (86.8%)	1,294 (82.2%)	
≤ 1	1,073 (13.9%)	811 (13.2%)	280 (17.8%)	
Marital status, *N* (%)				< 0.0001
Married/cohabiting	4,909 (63.6%)	3,951 (64.3%)	941 (59.8%)	
Never married	1,367 (17.7%)	1,180 (19.2%)	161 (10.2%)	
Widowed/divorced/separated	1,443 (18.7%)	1,014 (16.5%)	472 (30.0%)	
Education, *N* (%)				< 0.0001
College or above	1,250 (16.2%)	1,084 (16.9%)	198 (12.6%)	
High school or equivalent	2,980 (22.4%)	1,438 (22.4%)	353 (22.4%)	
Less than high school	3,288 (42.6%)	2,662 (41.5%)	765 (48.6%)	
More than high school	1,451 (18.8%)	1,231 (19.2%)	258 (16.4%)	
Smoke, *N* (%)				0.34
No	4,161 (53.9%)	3,398 (55.3%)	729 (46.3%)	
Yes	3,558 (46.1%)	2,747 (44.7%)	845 (53.7%)	
Drink status, *N* (%)				< 0.0001
Ever drink	826 (10.7%)	602 (9.8%)	246 (15.6%)	
Never drink	6,893 (89.3%)	5,542 (90.2%)	1,328 (84.4%)	
Diabetes, *N* (%)				< 0.0001
DM	1,126 (14.6%)	744 (12.1%)	438 (27.8%)	
IFG	316 (4.1%)	240 (3.9%)	80 (5.1%)	
IGT	301 (3.9%)	215 (3.5%)	87 (5.5%)	
No	5,971 (77.4%)	4,946 (80.5%)	969 (61.6%)	
Stroke, *N* (%)				< 0.0001
No	7,513 (97.4%)	6,034 (98.2%)	1,462 (92.9%)	
Yes	201 (2.6%)	111 (1.8%)	112 (7.1%)	
Hypertension, *N* (%)				< 0.0001
No	4,829 (62.6%)	4,086 (66.5%)	661 (42.0%)	
Yes	2,885 (37.4%)	2059 (33.5%)	913 (58.0%)	
Urine Creatinine, mg/dL		112.0 (67.0,165.0)	101.0 (63.0,147.0)	< 0.0001

Categorical variables were presented as N (%). BMI, body mass index; *N*, numbers of subject; NHANES, National Health and Nutrition Examination Survey; PIR, Poverty Income Ratio.

### 3.2 Concentrations and correlations of heavy metals

Supplementary Table S1 illustrates how the concentrations of 12 metals are distributed in urine. Detection rates for each metal were above 80.0%, except for Ur, with a detection rate of 73.8%. Mo was found at the highest concentration, while both Mo and Cs were detected in nearly all participants. Compared to individuals without OAB, those with the condition had notably increased levels of Pb, Cd, and Cs (all *p*-values < 0.05).

The Pearson correlation coefficients (Supplementary Figure S2) indicated moderate correlations between the following pairs: Tl and Cs (*r* = 0.56), Cd and Pb (*r* = 0.39), Ba and Co (*r* = 0.38), Mo and Tu (*r* = 0.37), and As and Hg (*r* = 0.31). On the contrary, the correlations between the remaining metals were relatively weak.

### 3.3 Association between exposures and OAB using logistic regression

Adjusted multivariate logistic regression analysis considering relevant covariates (Table 2), revealed a substantial elevation in OAB risk for the highest quartile (Q4) of urinary Cd, Pb, Co, Sb, and Ur when compared to the lowest quartile (Q1), with odds ratios (OR) and 95% confidence intervals (CI) as follows: Cd (OR 2.21, 95%CI 1.69–2.89), Pb (OR 1.44, 95%CI 1.15–1.81), Co (OR 1.29 95%CI 1.02–1.63), Ur (OR 1.33 95%CI 1.05–1.69), and Sb (OR 1.47 95%CI 1.17–1.86). Each unit increase in Ln-Cd, Ln-Pb, Ln-Co, and Ln-Sb corresponded to a 33, 22, 10, and 23% rise in OAB risk, respectively, with all *p*-values being less than 0.05. Additionally, a subgroup analysis was performed considering age and alcohol consumption (Table 2). The study identified a notable rise in OAB risk in the highest quartile (Q4) compared to the lowest quartile (Q1) of Cd (OR 2.74, 95% CI 1.94–3.87) and Pb (OR 1.84, 95% CI 1.35–2.52) among young and middle-aged individuals. The associations persisted as significant when analyzing Ln-transformed metal concentrations and OAB risk, with all *p*-values remaining below 0.05. In the ever-drinkers group, the risk of OAB was notably higher in the top quartile (Q4) compared to the bottom quartile (Q1) for urinary Cd with an odds ratio (OR) of 2.65 (95% confidence interval [CI]: 1.95–3.61), Pb with an OR of 1.58 (95% CI: 1.22–2.05), and Co with an OR of 1.36 (95% CI: 1.06–1.75). These associations maintained statistical significance even after considering the natural Ln-transformation of metal concentrations and the risk of OAB (all *p*-values < 0.05). In the older adult group, a strong positive relationship exists between OAB and urinary Ba was observed only in the third exposure quantile (Q3) (OR 0.72, 95% CI 0.55–0.94), with no correlation detected in the highest exposure quantile (Q4). Additionally, there were no significant correlations between other metals and OAB. In the never-drinkers group, there was a notable rise in OAB risk in the second quartile (Q2) of urinary cadmium (Cd) (OR 0.50, 95% CI 0.29–0.86) and Hg (OR 2.05, 95% CI 1.15–3.65), but this increase was not observed in the fourth quartile (Q4). No notable correlations were identified between other metals and OAB.

**Table 2:** Associations of single urinary metals with OAB risk in the NHANES 2005–2016 cycles.

Metals (μg/g creatinine)	Q1	Q2		Q3		Q4		Continuous	
		OR (95% CI)	*p* value	OR (95% CI)	*p* value	OR (95% CI)	*p* value	OR (95% CI)	*p* value
Ba									
Overall	Ref	0.92 (0.72, 1.17)	0.47	0.78 (0.60, 1.02)	0.07	0.81 (0.63, 1.05)	0.12	0.91 (0.82, 1.00)	0.06
Age (20–59)		0.97 (0.68, 1.38)	0.87	0.90 (0.63, 1.29)	0.56	0.83 (0.59, 1.18)	0.30	0.89 (0.78, 1.02)	0.09
Age ≥ 60		0.88 (0.65, 1.19)	0.41	0.72 (0.55, 0.94) *	0.02	0.83 (0.60, 1.16)	0.28	0.94 (0.82, 1.07)	0.34
Ever drink		0.85 (0.65, 1.10)	0.20	0.78 (0.58, 1.04)	0.09	0.80 (0.60, 1.06)	0.11	0.91 (0.81, 1.02)	0.10
Never drink		1.65 (1.00, 2.73)	0.05	0.97 (0.56, 1.68)	0.90	1.10 (0.62, 1.97)	0.73	0.99 (0.80, 1.24)	0.94
Cd									
Overall	Ref	1.59 (1.22, 2.07) *	<0.001	1.91 (1.47, 2.48) *	<0.0001	2.21 (1.69, 2.89) *	<0.0001	1.33 (1.20, 1.46) *	<0.0001
Age (20–59)		1.65 (1.15, 2.38) *	0.01	1.67 (1.16, 2.40) *	0.01	2.74 (1.94, 3.87) *	<0.0001	1.42 (1.24, 1.62) *	<0.0001
Age ≥ 60		1.16 (0.84, 1.61)	0.37	1.28 (0.91, 1.80)	0.16	1.12 (0.84, 1.49)	0.34	1.08 (0.92, 1.26)	0.34
Ever drink		1.92 (1.40, 2.62) *	<0.0001	2.31 (1.71, 3.11) *	<0.0001	2.65 (1.95, 3.61) *	<0.0001	1.39 (1.25, 1.55) *	<0.0001
Never drink		0.50 (0.29, 0.86) *	0.01	0.94 (0.50, 1.75)	0.83	0.79 (0.40, 1.55)	0.49	0.98 (0.72, 1.35)	0.92
Pb									
Overall	Ref	1.15 (0.91,1.44) *	0.24	1.30 (1.05,1.62) *	0.02	1.44 (1.15,1.81) *	0.002	1.22 (1.11,1.35) *	<0.001
Age (20–59)		1.08 (0.73, 1.58)	0.70	1.16 (0.84, 1.59)	0.36	1.84 (1.35, 2.52) *	<0.001	1.34 (1.16, 1.55) *	<0.001
Age ≥ 60		0.94 (0.67, 1.32)	0.72	1.15 (0.84, 1.58)	0.38	0.92 (0.61, 1.40)	0.71	0.98 (0.79, 1.21)	0.83
Ever drink		1.16 (0.88, 1.51)	0.28	1.30 (1.01, 1.68) *	0.04	1.58 (1.22, 2.05) *	<0.001	1.26 (1.13, 1.40) *	<0.0001
Never drink		1.23 (0.78, 1.93)	0.37	1.28 (0.78, 2.10)	0.33	1.11 (0.64, 1.92)	0.72	1.10 (0.83, 1.45)	0.52
Hg									
Overall	Ref	1.01 (0.80, 1.26)	0.96	1.16 (0.93, 1.44)	0.20	0.94 (0.74, 1.21)	0.64	0.98 (0.90, 1.07)	0.67
Age (20–59)		1.05 (0.77, 1.42)	0.76	1.05 (0.75, 1.49)	0.76	0.88 (0.59, 1.30)	0.51	0.94 (0.82, 1.08)	0.36
Age ≥ 60		0.92 (0.67, 1.26)	0.59	1.16 (0.82, 1.65)	0.39	0.96 (0.68, 1.34)	0.80	1.01 (0.89, 1.14)	0.93
Ever drink		0.90 (0.71, 1.15)	0.39	1.07 (0.84, 1.37)	0.57	0.95 (0.73, 1.23)	0.68	0.98 (0.89, 1.08)	0.68
Never drink		2.05 (1.15, 3.65) *	0.02	1.96 (1.00, 3.43)	0.05	1.09 (0.59, 2.03)	0.78	1.06 (0.89, 1.27)	0.52
Cs									
Overall	Ref	0.85 (0.68, 1.05)	0.13	1.05 (0.85, 1.30)	0.64	1.07 (0.86, 1.34)	0.55	1.08 (0.90, 1.29)	0.39
Age (20–59)		0.72 (0.50, 1.02)	0.06	0.88 (0.64, 1.21)	0.44	1.01 (0.71, 1.44)	0.96	1.10 (0.85, 1.42)	0.45
Age ≥ 60		0.98 (0.75, 1.27)	0.88	1.09 (0.78, 1.53)	0.60	1.08 (0.80, 1.44)	0.62	1.00 (0.79, 1.26)	0.97
Ever drink		0.93 (0.73, 1.18)	0.53	1.19 (0.95, 1.50)	0.12	1.15 (0.89, 1.48)	0.28	1.14 (0.93, 1.38)	0.20
Never drink		0.50 (0.26, 0.97) *	0.04	0.60 (0.33, 1.07)	0.08	0.72 (0.38, 1.35)	0.30	0.80 (0.52, 1.25)	0.32
Co									
Overall	Ref	1.03 (0.77, 1.37)	0.84	1.15 (0.87, 1.52)	0.31	1.29 (1.02, 1.63) *	0.03	1.10 (1.00, 1.24)	0.04 *
Age (20–59)		1.01 (0.72, 1.42)	0.96	1.35 (0.96, 1.92)	0.09	1.21 (0.85, 1.72)	0.28	1.08 (0.89, 1.30)	0.45
Age ≥ 60		1.08 (0.75, 1.55)	0.69	0.88 (0.62, 1.24)	0.45	1.37 (1.01, 1.87)	0.07	1.09 (0.94, 1.26)	0.24
Ever drink		1.09 (0.81, 1.45)	0.56	1.13 (0.84, 1.54)	0.41	1.36 (1.06, 1.75) *	0.02	1.09 (1.01, 1.25)	0.03 *
Never drink		1.22 (0.71, 2.10)	0.47	1.34 (0.83, 2.14)	0.22	1.29 (0.74, 2.23)	0.36	1.17 (0.87, 1.56)	0.30
Mo									
Overall	Ref	0.98 (0.78, 1.22)	0.84	1.06 (0.85, 1.31)	0.62	1.04 (0.84, 1.29)	0.71	1.01 (0.89, 1.16)	0.85
Age (20–59)		1.07 (0.79, 1.44)	0.67	0.91 (0.65, 1.28)	0.58	0.98 (0.70, 1.38)	0.93	0.92 (0.75, 1.13)	0.41
Age ≥ 60		0.93 (0.69, 1.26)	0.63	1.23 (0.92, 1.63)	0.15	1.17 (0.87, 1.58)	0.29	1.15 (0.98, 1.35)	0.09
Ever drink		0.95 (0.75, 1.21)	0.69	0.98 (0.77, 1.24)	0.86	1.05 (0.82, 1.34)	0.69	1.02 (0.89, 1.17)	0.78
Never drink		0.81 (0.47, 1.38)	0.43	0.99 (0.62, 1.58)	0.96	1.49 (0.82, 2.73)	0.19	1.01 (0.70, 1.46)	0.95
Sb									
Overall	Ref	1.26 (1.01, 1.58) *	0.04	1.41 (1.13, 1.75) *	0.003	1.47 (1.17, 1.86) *	0.002	1.23 (1.10, 1.37) *	<0.001
Age (20–59)		1.26 (0.93, 1.70)	0.14	1.31 (0.96, 1.79)	0.09	1.30 (0.96, 1.77)	0.09	1.16 (1.00, 1.35) *	0.049
Age ≥ 60		1.33 (0.95, 1.87)	0.09	1.51 (0.99, 2.11)	0.07	1.71 (0.98, 2.38)	0.06	1.31 (0.89, 1.57)	0.212
Ever drink		1.43 (1.14, 1.79) *	0.003	1.55 (1.21, 1.97) *	<0.001	1.54 (1.20, 1.97) *	<0.001	1.22 (1.08, 1.37) *	0.001
Never drink		0.90 (0.49, 1.67)	0.74	0.93 (0.59, 1.47)	0.76	1.37 (0.83, 2.24)	0.21	1.33 (1.01, 1.75) *	0.04
Tl									
Overall	Ref	0.95 (0.77, 1.17)	0.62	0.81 (0.64, 1.04)	0.10	0.99 (0.81, 1.20)	0.90	0.96 (0.83, 1.12)	0.60
Age (20–59)		0.96 (0.72, 1.27)	0.75	0.79 (0.56, 1.12)	0.18	0.94 (0.70, 1.27)	0.68	0.94 (0.77, 1.17)	0.59
Age ≥ 60		0.84 (0.58, 1.22)	0.36	0.85 (0.61, 1.20)	0.36	0.96 (0.67, 1.37)	0.82	1.00 (0.80, 1.24)	1.00
Ever drink		1.05 (0.84, 1.31)	0.66	0.86 (0.66, 1.12)	0.27	1.03 (0.82, 1.30)	0.80	0.99 (0.83, 1.17)	0.87
Never drink		0.68 (0.37, 1.28)	0.23	0.86 (0.49, 1.51)	0.59	1.03 (0.55, 1.95)	0.92	0.88 (0.59, 1.32)	0.54
Tu									
Overall	Ref	0.85 (0.66, 1.09)	0.19	0.82 (0.65, 1.04)	0.11	1.13 (0.89, 1.43)	0.32	1.06 (0.95, 1.18)	0.31
Age (20–59)		0.71 (0.49, 1.05)	0.09	0.66 (0.46, 0.97)	0.03	0.99 (0.70, 1.40)	0.95	1.03 (0.86, 1.23)	0.78
Age ≥ 60		1.13 (0.79, 1.60)	0.50	1.09 (0.77, 1.55)	0.62	1.40 (1.02, 1.91)	0.04	1.10 (0.95, 1.26)	0.19
Ever drink		0.83 (0.61, 1.13)	0.24	0.81 (0.61, 1.08)	0.15	1.10 (0.83, 1.47)	0.49	1.06 (0.93, 1.20)	0.37
Never drink		0.98 (0.55, 1.76)	0.94	0.89 (0.55, 1.44)	0.62	1.36 (0.73, 2.52)	0.32	1.06 (0.84, 1.35)	0.60
As									
Overall	Ref	1.01 (0.83, 1.24)	0.89	1.07 (0.87, 1.31)	0.52	0.92 (0.73, 1.15)	0.44	0.99 (0.91, 1.07)	0.73
Age (20–59)		1.05 (0.78, 1.42)	0.74	0.87 (0.63, 1.21)	0.40	0.85 (0.63, 1.15)	0.30	0.93 (0.83, 1.05)	0.22
Age ≥ 60		1.08 (0.78, 1.49)	0.64	1.38 (1.01, 1.89)	0.04	1.02 (0.73, 1.45)	0.89	1.04 (0.93, 1.16)	0.50
Ever drink		1.06 (0.84, 1.33)	0.64	1.07 (0.86, 1.35)	0.53	0.94 (0.73, 1.20)	0.60	0.99 (0.90, 1.08)	0.79
Never drink		0.80 (0.43, 1.50)	0.49	1.12 (0.64, 1.98)	0.68	0.90 (0.51, 1.56)	0.70	0.99 (0.82, 1.20)	0.95
Ur									
Overall	Ref	1.14 (0.91, 1.44)	0.26	1.15 (0.88, 1.49)	0.31	1.33 (1.05, 1.69) *	0.02	1.09 (0.99, 1.19)	0.08
Age (20–59)		1.22 (0.86, 1.72)	0.25	1.10 (0.75, 1.61)	0.62	1.38 (0.99, 1.91)	0.06	1.10 (0.98, 1.23)	0.12
Age ≥ 60		1.31 (0.94, 1.81)	0.10	1.27 (0.89, 1.80)	0.18	1.15 (0.82, 1.63)	0.41	1.06 (0.92, 1.21)	0.42
Ever drink		1.15 (0.89, 1.50)	0.28	1.13 (0.84, 1.54)	0.41	1.27 (0.98, 1.63)	0.07	1.07 (0.97, 1.19)	0.16
Never drink		1.27 (0.73, 2.19)	0.39	1.30 (0.65, 2.58)	0.45	1.85 (1.00, 3.33)	0.05	1.19 (0.97, 1.45)	0.10

All models were adjusted for age, sex, race/ethnicity, education, Poverty Income Ratio, marital status, body mass index, smoke, drinking status, diabetes, stroke, hypertension, urine creatinine and NHANES cycles. Continuous, Ln-transformed concentration of metal; CI: confidence interval; OR: odds ratio; Q, quartile; Ref, reference.

To tackle confounding effects in this sensitivity analysis, logistic regression models included all urinary metals. The study found that individuals in the highest quartile (Q4) of urinary Cd, Co, Sb, and Pb had a significantly increased risk of overactive bladder (OAB), with OR and 95% CI of 2.01 (1.51–2.67), 1.34 (1.06–1.69), 1.36 (1.04–1.79), and 1.24 (1.01–1.61), respectively. Conversely, the fourth quartile of urinary Ba (OR: 0.70, 95% CI: 0.53–0.92) decreased the risk. Each unit increase in Ln-Cd, Ln-Pb, Ln-Co, and Ln-Sb enhanced the risk by 28.0, 14.0, 11.0, and 17%, respectively, while each unit increase in Ln-Ba decreased the risk by 14% (all *p*-values <0.05) (Supplementary Table S2).

### 3.4 Association between exposures and OAB using WQS

**Figure 2:**
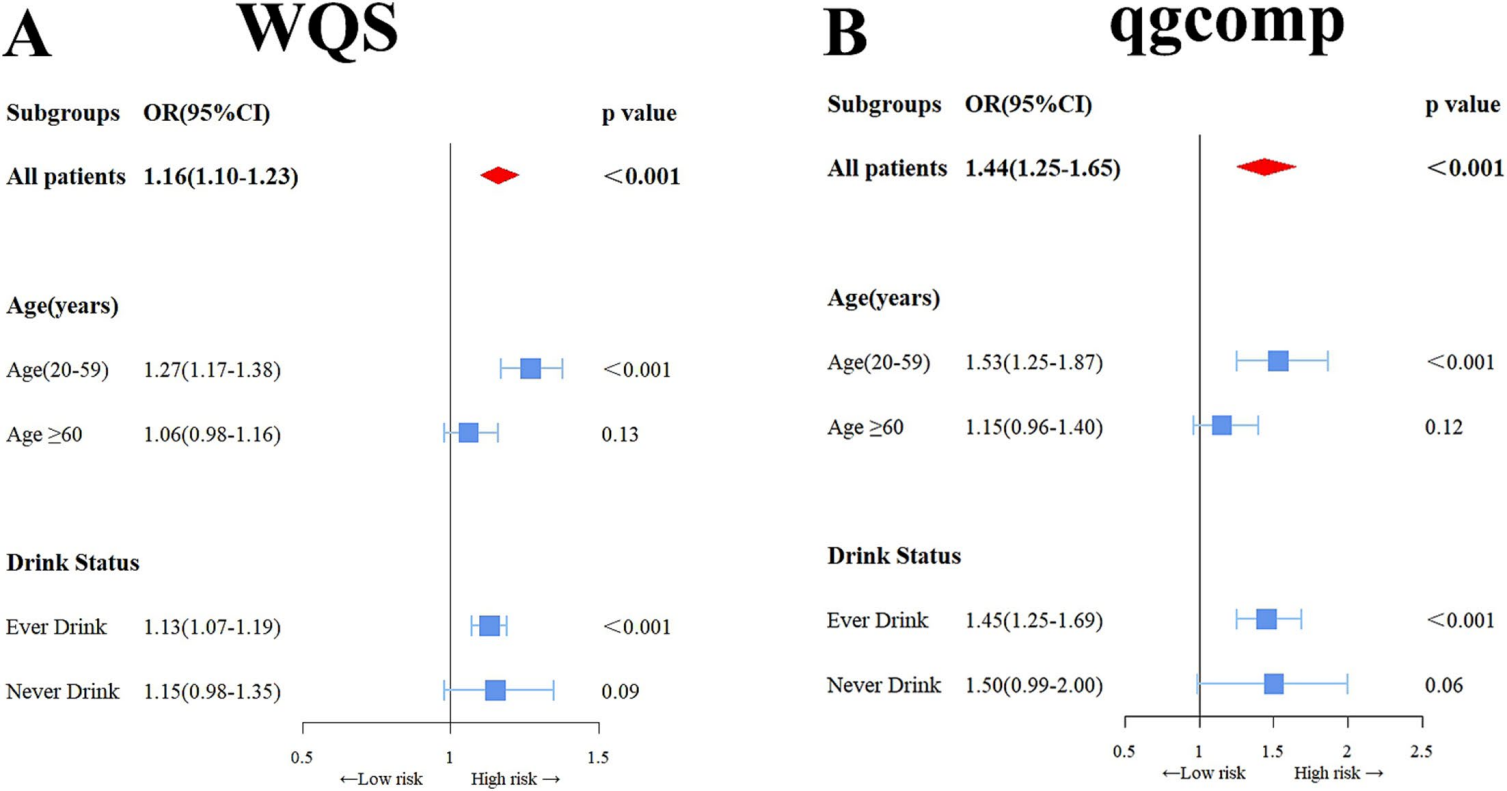
Odds ratios (95% confidence intervals, CI) depicting the association of OAB with co-exposure to urinary metal mixtures, were analyzed using weighted quantile sum (WQS) **(A)** and qgcomp **(B)** methods. Adjusted for age, sex, race/ethnicity, education, Poverty Income Ratio, marital status, body mass index, smoking habit, alcohol consumption, diabetes, stroke, hypertension, urine creatinine, and NHANES cycles.

**Figure 3:**
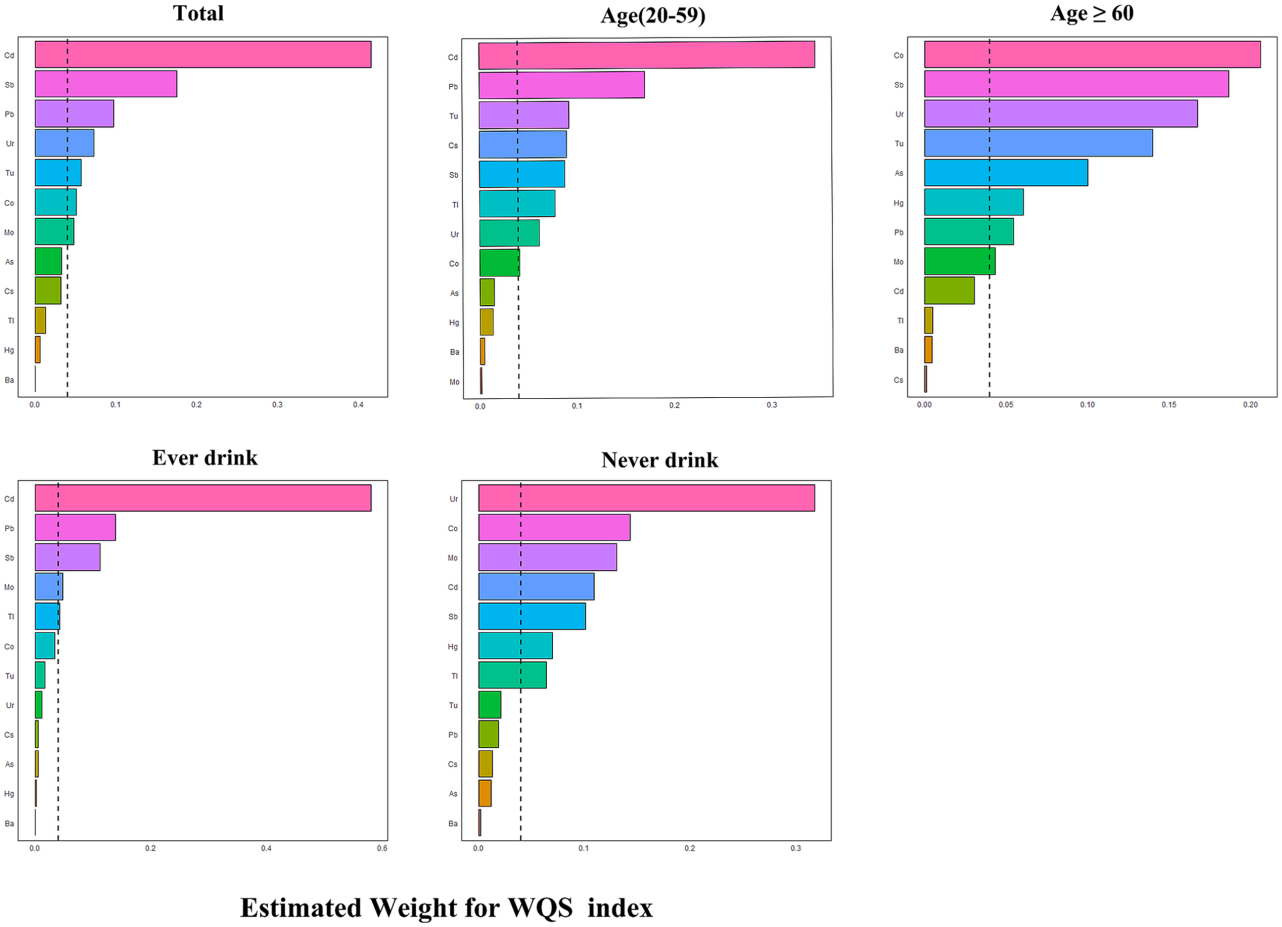
Estimated weights of metals for OAB by WQS models adjusted for age, sex, race/ethnicity, education, Poverty Income Ratio, marital status, body mass index, smoke, drinking status, diabetes, stroke, hypertension, urine creatinine and NHANES cycles.

Figure 2A in the study shows a notable rise in the risk of OAB linked to the WQS index of urinary metals (OR: 1.16, 95% CI: 1.10–1.23), showing that being exposed to urinary metals together has a stimulating effect on OAB. Urinary metals and OAB were shown in the subgroup analysis, which was stratified by age and alcohol consumption, which were strongly positively correlated in the young and middle-aged group and ever-drinkers group (OR: 1.53, 95% CI: 1.25–1.87) and (OR: 1.45, 95% CI: 1.25–1.69), respectively. Nevertheless, this correlation did not reach statistical significance in the older adult and never-drinkers groups (OR: 1.15, 95% CI: 0.96–1.40) and (OR: 1.45, 95% CI: 0.99–2.00). The weight estimation for each WQS index is illustrated in Figure 3. In urinary mixtures, Cd displayed the highest weight across the entire population, as well as in the young and middle-aged groups and the ever-drinkers group. In contrast, Co and Ur were identified as the predominant weighted metals in the older adult group and the never-drinkers group, respectively.

In the sensitivity analysis using the qgcomp model, the joint exposure to metals in urine was still significantly linked to OAB. in the total population (OR: 1.44, 95% CI: 1.25–1.65), as well as in the young and middle-aged group (OR: 1.53, 95% CI: 1.25–1.87) and the ever-drinkers group (OR: 1.45, 95% CI: 1.25–1.69). No notable link was observed. in the older adult group (OR: 1.014, 95% CI: 0.996–1.032) and the never-drinkers group (OR: 1.50, 95% CI: 0.99–2.00) (Figure 2B). In Supplementary Figure S3, the weights, both positive and negative, for each factor are illustrated, revealing Cd and Co as the primary positive drivers within the urinary metal mixtures.

### 3.5 Association between exposures and OAB using BKMR

**Figure 4:**
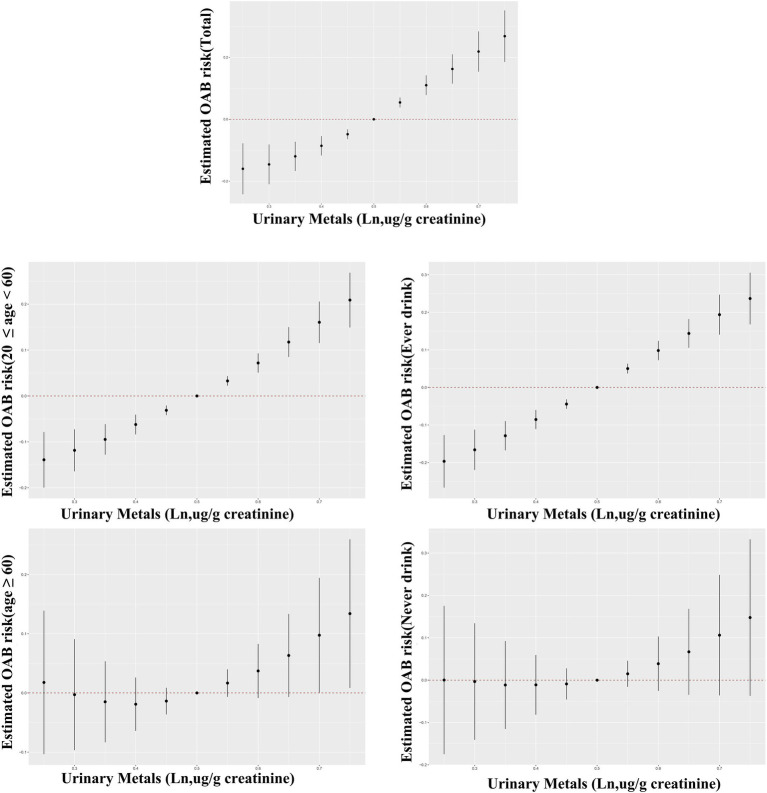
Bayesian Kernel Machine Regression (BKMR) models illustrate the joint effects of urinary metal mixtures on OAB risk in the total population and subgroups. The comparison involves all metals at specific percentiles versus their 50th percentile. The models were adjusted for age, sex, race/ethnicity, education, poverty-income ratio, marital status, body mass index, smoking habit, alcohol consumption, diabetes, stroke, hypertension, urine creatinine, and NHANES cycles.

**Figure 5:**
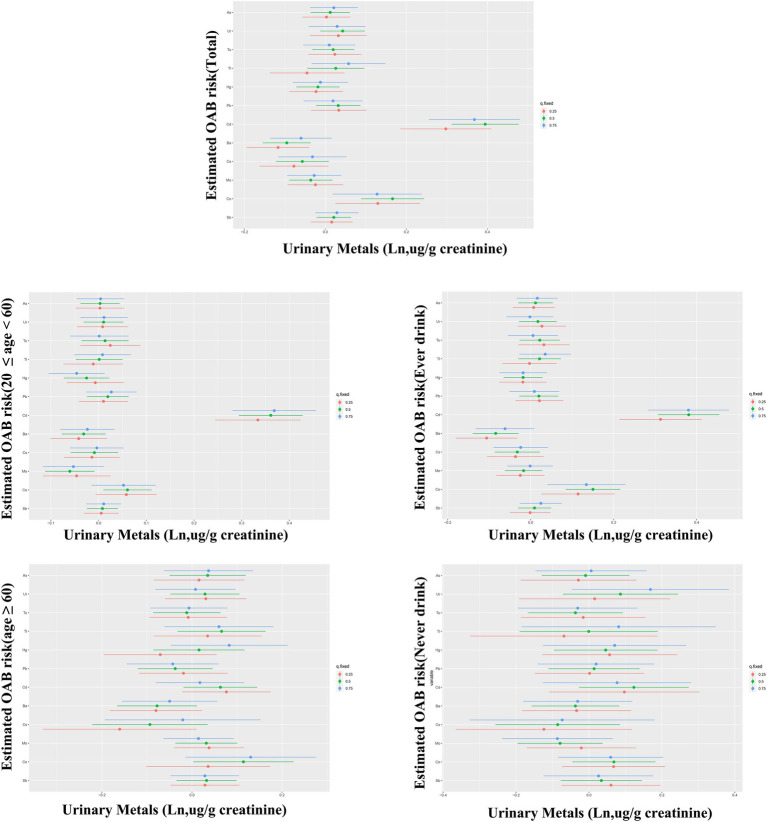
Bayesian kernel machine regression (BKMR) models showcase the associations of single urinary metals with OAB risk in the total population and subgroups. Metals are evaluated when all other metals are held at corresponding 25th (red), 50th (green), or 75th (blue). The models were adjusted for age, sex, race/ethnicity, education, poverty-income ratio, marital status, body mass index, smoking habit, alcohol consumption, diabetes, stroke, hypertension, urine creatinine, and NHANES cycles.

**Figure 6:**
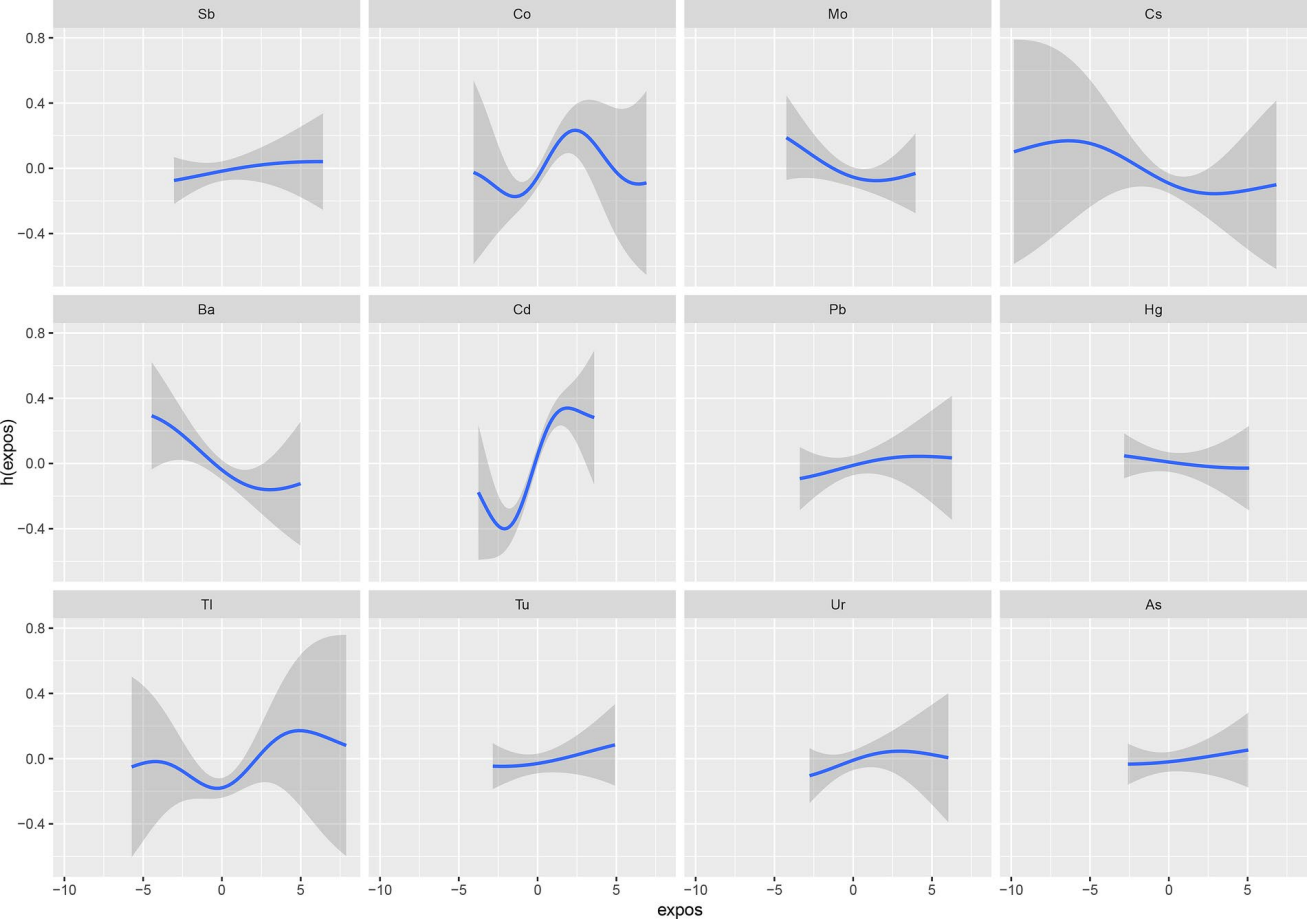
The interaction of urinary metals for psoriasis by BKMR models adjusted for age, sex, race/ethnicity, education, Poverty Income Ratio, marital status, body mass index, smoke, drinking status, diabetes, stroke, hypertension, urine creatinine, and NHANES cycles.

The BKMR analysis revealed a notably stronger overall effect in the 50th–75th percentile than in the 25th–50th percentile across the total population, young and middle-aged individuals, and ever-drinkers (Figure 4). When urinary metal concentrations were controlled at the 25th, 50th, and 75th percentiles, Cd and Co significantly increased OAB risk in the overall population and among ever-drinkers. Additionally, Co had a notable impact on OAB risk at the 50th percentile in the young and middle-aged group (Figure 5 and Supplementary Table S3). Posterior inclusion probabilities for urinary metals exceeded 0.50 across all groups. Cd ranked highest in the total population, young and middle-aged group, and ever-drinkers group. Cesium (Cs) and Thallium (Tl) ranked highest in the young and middle-aged group and never-drinkers group, respectively. Considering the moderate correlation among certain urinary metals, 12 urinary metal interactions were further investigated separately, indicating possible interactions among certain metals found in urine (such as urinary Tl and Cs; urinary Ba and Co; urinary Cd and Pb) in individuals with OAB (Figure 6 and Supplementary Figure S4).

**Figure 7:**
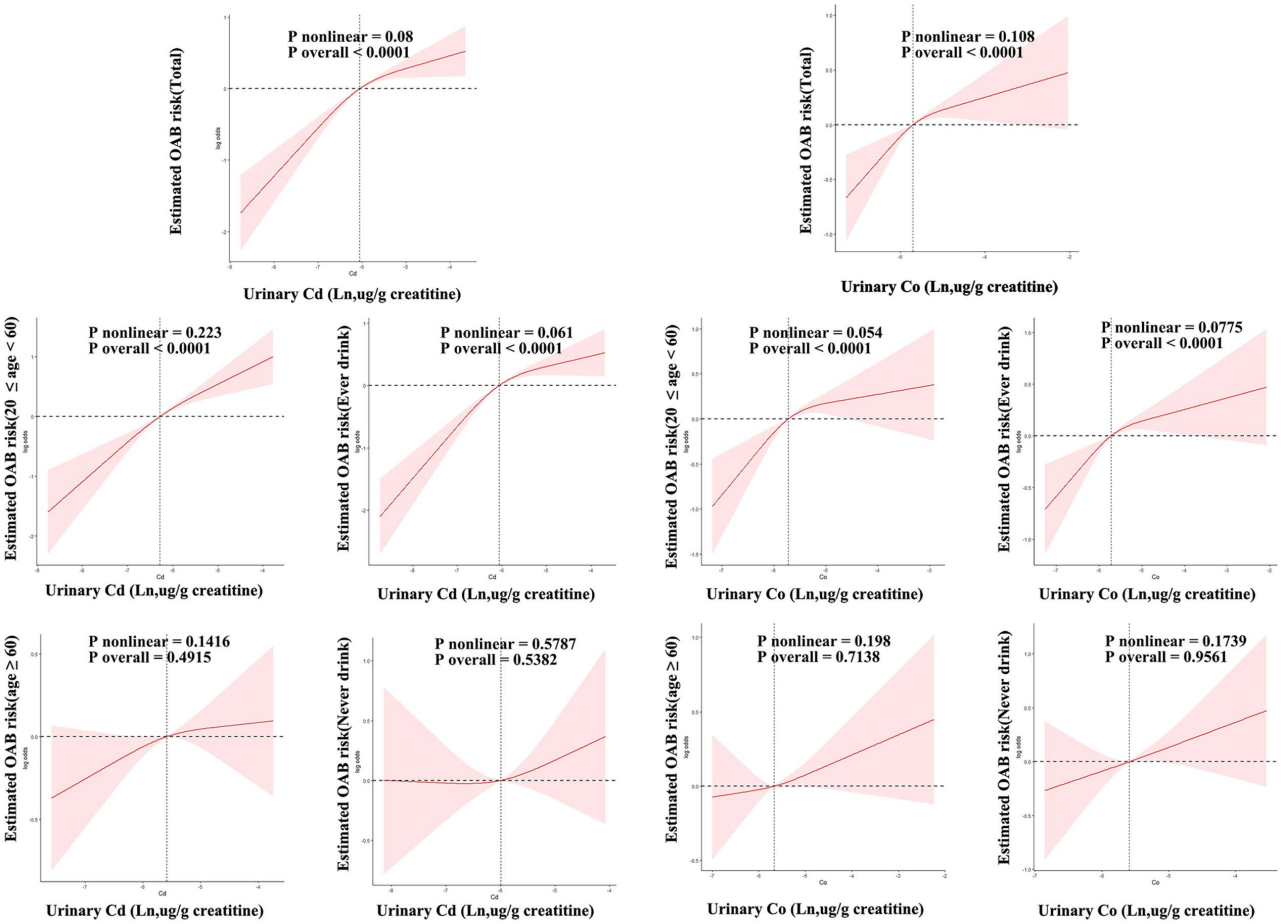
Restricted cubic spline (RCS) models unveil the dose–response relationship between urinary cadmium (Cd), Co, and OAB risk. The models were adjusted for age, sex, race/ethnicity, education, poverty-income ratio, marital status, body mass index, smoking habit, alcohol consumption, diabetes, stroke, hypertension, urine creatinine, and NHANES cycles. The solid line signifies odds ratios, while the red-shaded area signifies the 95% CI.

Figure 7 shows how urinary levels of Cd and Co correlate with the risk of OAB, as determined by RCS regression. The study found a linear relationship between Cd and Co concentrations and OAB risk, indicating that higher urinary levels of Cd (nonlinear *p*-value = 0.08) and Co (nonlinear *p*-value = 0.108) are associated with an increased risk of OAB (overall *p*-value = 0.001 for both). A comparable dose–response relationship was found. Across the young, middle-aged, and ever-drinkers groups, with significant overall *p*-values for both Cd and Co (overall *p*-value < 0.001) and non-significant nonlinear p-values (young and middle-aged: Cd = 0.223, Co = 0.054; ever-drinkers: Cd = 0.061, Co = 0.0775). However, this association was not evident in the older adult and never-drinkers groups.

## 4 Discussion

This study, using a cross-sectional design, is the first to deeply analyze the combined influence of urinary metal mixtures on OAB risk within a broad, nationally representative sample. Employing multiple statistical methods, our findings reveal that individuals diagnosed with OAB exhibited significantly increased urinary Cd, Co, and Pb concentrations compared with those without OAB. Single logistic regression models for individual metals found that urinary levels of Cd, Pb, Co, and Sb are independent risk factors for OAB. Logistic regression models demonstrated a meaningful positive connection for multiple metals. Between urinary Cd and Co with OAB risk, while Ba demonstrated a protective effect against OAB. A consistent significant positive association between co-exposure to urinary metals and OAB risk was shown by both WQS regression and BKMR models, which also identifying Ba as the primary contributing factor. Further evidence for these findings was provided by the qgcomp model and RCS regression analysis. A systematic review by (37) identified several pathophysiological phenotypes of overactive bladder (OAB), such as detrusor and urothelial dysfunctions, autonomic nervous system dysfunction, metabolic syndrome, sex hormone deficiency, and changes in urinary microbiota.

Cd, a transition metal, has seen a significant increase in industrial usage in recent years, particularly in batteries, alloys, and pigments. Cd exposure primarily occurs through food consumption as a byproduct during the extraction of other metals from ores (15). Cd can induce OAB through direct interactions with the urinary system. While the kidney is the primary target of Cd toxicity (38), the formation of Cd-protein complexes, especially with metallothionein, can undergo glomerular filtration and persist within the bladder for extended durations, contributing to tissue damage (39). Further investigation with longitudinal methods and a range of populations is essential for improved comprehension for the complex relationships between heavy metal exposure and OAB. Additionally, particular urinary metals found in this research might provide important understanding of the mechanisms behind OAB development. The potential role of cadmium in bladder cancer development was investigated in 2019 (40). According to their findings, Cd may be involved in the disruption of bladder function. Animal experiments have further demonstrated that exposure to Cd2 + for 3 months can adversely affect the neurogenic and myogenic contraction activities of the rat detrusor muscle, potentially leading to the manifestation of OAB (41).

Current research on OAB predominantly explores neurological factors, with anticholinergic drugs playing a pivotal role in treatment. The initiation of urination involves the pontine micturition center stimulating the release of acetylcholine, activating nicotinic receptors on the external urethral sphincter’s striated muscle. Prolonged exposure to Cd in mice through diet influences acetylcholinesterase activity in adult rodents’ brain areas (42). Simultaneously, Cd may impede the release of acetylcholine (ACh) by interfering with calcium metabolism (43), potentially resulting in abnormal urination and, consequently, OAB. In this research, we identified a positive correlation between urinary Cd and the risk of OAB. Furthermore, it was also found that there is a negative correlation between Ba and the risk of OAB development. The mechanisms of Ba’s urinary system toxicity remain unclear. Potential mechanisms include the regulation of oxidative stress and cellular apoptosis, both of which significantly influence the typical progression of neurogenesis (44).

In addition, there was a positive association between the risk of OAB and certain toxic metals, including Co, Pb, and Sb. Co and its compounds are ubiquitously present in nature and contribute to several anthropogenic activities. Cobalt is important for vitamin B12, but overexposure can result in harmful health effects (45). Additionally, Co compounds have cytotoxic and genotoxic effects on human urothelial cells, and solubility may be a crucial component of Co-induced toxicity (46). Additionally, Co induces neurodegenerative damage by disrupting autophagic flux through the activation of hypoxia-inducible factor-1α, leading to nervous system dysfunction (47). Pb exposure persists due to legacy uses such as plumbing and household paints, current industrial activities like battery manufacturing, extensive soil contamination, and its presence in tobacco and tobacco smoke (48). The neurotoxic effects of Pb involve mechanisms such as cytotoxicity (49). Research found that Pb and Cd had a synergistic effect on adult males exposed to these metals at work in terms of their serum testosterone levels. This suggests that the interaction may lead to sex hormone deficiency and OAB (50). An increase has been observed in the concentration of Sb in the ecological environment (51). Antimony has been shown in cell experiments to trigger autophagy via reactive oxygen species, which mediates cytotoxicity and ultimately results in neuronal apoptosis (52). It is imperative to increase awareness of the effects of heavy metal exposure in the general population and look into the mechanisms that underlie the risk of OAB.

Now it is unclear how metal mixtures work together to affect OAB. According to research, oxidative stress, hematological changes, and a variety of adverse health outcomes are linked to concurrent exposure to multiple heavy metals (53, 54). To address this knowledge gap, we investigated the combined effects of several metals on OAB by using newly developed analytical methods. Recent data points to demographic differences in OAB prevalence, pathophysiology, and clinical patterns, including differences among young, middle-aged, older adult, ever-drinkers, and never-drinker’s groups (55–57). Subgroup analyses were consequently conducted based on age and alcohol consumption. The WQS model was specifically developed to evaluate co-exposure effects by identifying the relative significance of individual metals in mixtures. Furthermore, the BKMR model proved effective in discerning nonlinear and non-additive connections among metals.

In this study, the WQS regression findings closely matched those from the BKMR model. Urinary metal mixtures, especially Cd, were associated with a higher risk of OAB, according to the analysis. The positive association was more significant in young and middle-aged individuals and ever-drinkers than in the older adult and never-drinkers. Cd was the most significant metal in the young and middle-aged and ever-drinker’s groups, whereas Tl was predominant in the older adult and never-drinker’s groups. The observed discrepancy might result from higher levels of heavy metal exposure at work among young and middle-aged individuals. More than 500,000 Americans, according to the Agency for Toxic Substances and Disease Registry may be exposed to occupational cadmium, mainly through inhaling dust and airborne particles from smelting operations (58, 59).

Moreover, individuals who regularly consume alcohol may face elevated Cd exposure due to the substantial Cd content present in alcoholic beverages like wine and beer (60). Alcohol and Cd exposure together increase the body’s ability to accumulate Cd, according to a number of toxicological studies conducted on rats (61, 62). In addition, Cd disrupts calcium signaling in cells (63). The human body primarily absorbs Tl and its compounds through skin contact, drinking water, breathing in ambient air, and eating tainted foods like lettuce and cabbage. The impact may be more significant for older individuals due to Tl accumulation in the food chain (64); though the relationship between Tl and alcohol is still uncertain. Using the BKMR model, the complex relationship between particular urinary metals was examined. Studies indicate that exposure to Pb, Cd, As, and Hg in the environment may cause synergistic neurotoxicity in humans (65). Additionally, It has been shown that co-exposure to a combination of Pb, As, and Mn changes the cholinergic system (66), potentially contributing to bladder dysfunction. Sensitivity analyses, including the application of qgcomp and RCS models, were conducted to ensure result reliability. It also uses multiple imputation showed similar results, suggesting minimal bias. However, non-random missingness (For instance, healthier participants being more likely to complete surveys) could underestimate associations. The qgcomp regression analysis highlighted the harmful effects of urinary metal mixtures on OAB, identifying Cd and Co as the main positive contributors. Urinary levels of Cd and Co were directly linearly correlated with the risk of OAB, according to RCS analysis. In addition, the stronger associations in young/middle-aged adults and ever-drinkers may reflect higher cumulative metal exposure [occupational activity (67), alcohol-related Cd uptake (68)] or reduced detoxification capacity compared to older adults.

This study presented several notable advantages. This study’s strengths include a thorough evaluation of heavy metal exposure’s impact on OAB risk and the use of advanced statistical analyses within a large, diverse population. The consistent results from various perspectives enhance the reliability of the findings. However, it’s crucial to acknowledge certain limitations. The cross-sectional design of the study restricts the ability to draw conclusions about causality, and recall bias may be introduced by using self-reported OAB diagnoses. And the cross-sectional design limits causal inference between heavy metal exposure and OAB risk, as temporal relationships cannot be established. Unmeasured confounders (e.g., dietary habits, occupational exposures, or genetic predispositions) may influence both metal accumulation and OAB development. For example, individuals with higher metal exposure might also exhibit lifestyle factors (e.g., smoking) that independently contribute to OAB. Accurately measuring metal concentrations remains challenging due to varying half-lives in the body. Rigorous prospective studies with larger samples are imperative to validate these associations.

## 5 Conclusion

In summary, this cross-sectional study illuminates metals such as urinary Cd, Co, Pb, and Sb significantly linked to increased OAB risk, while Ba emerges as a protective factor. Consistent positive correlations in metal co-exposure analyses, with Cd and Co as primary contributors, were noted in both young middle-aged and ever-drinkers groups. Despite study limitations, the findings provide valuable insights, emphasizing the need for rigorous prospective studies to validate these associations further.

## Data Availability

The original contributions presented in the study are included in the article/Supplementary material, further inquiries can be directed to the corresponding authors.
