# Supplementary material for: Association between urinary heavy metal mixtures and overactive bladder risk in the U.S. adult population: a cross-sectional study
Source: Front Public Health. 2025 Nov 13;13:1577413. doi: 10.3389/fpubh.2025.1577413 (PMC12657428; doi:10.3389/fpubh.2025.1577413)
Supplement: Supplementary file 1 [file Table_1.docx]

**Table S1.** The distributions of 12 metal concentrations in urine in the NHANES 2005-2016 cycles

|  |  |  | Total (n = 7719) | | | |  | Without OAB (n = 6145) | | | |  | OAB (n = 1574) | | | |  |
| --- | --- | --- | --- | --- | --- | --- | --- | --- | --- | --- | --- | --- | --- | --- | --- | --- | --- |
| Metals | N | Detection  rates (%) | Median (IQR) | Min | Mean | Max |  | Median (IQR) | Min | Mean | Max |  | Median (IQR) | Min | Mean | Max | *p* value |
| **Urine (μg/g creatinine)** |  |  |  |  |  |  |  |  |  |  |  |  |  |  |  |  |  |
| Sb | 7719 | 96.6 | 0.07(0.01) | 0.07 | 0.07 | 3.51 |  | 0.07(0.01) | 0.01 | 0.07 | 2.24 |  | 0.08 (0.01) | 0.00 | 0.34 | 3.52 | 0.12 |
| Co | 7719 | 97.3 | 0.50(0.02) | 0.04 | 0.50 | 34.76 |  | 0.49(0.02) | 0.04 | 0.50 | 33.77 |  | 0.55(0.03) | 0.07 | 0.44 | 34.76 | 0.03 |
| Cs | 7719 | 100.0 | 4.95(0.06) | 0.73 | 4.77 | 111.75 |  | 4.90(0.07) | 0.97 | 4.77 | 111.75 |  | 5.21(0.13) | 0.73 | 4.77 | 28.54 | 0.02 |
| Ba | 7719 | 95.8 | 2.05(0.06) | 0.02 | 1.91 | 124.69 |  | 2.04(0.06) | 0.03 | 1.91 | 68.21 |  | 2.05(0.15) | 0.02 | 1.91 | 124.69 | 0.95 |
| Mo | 7719 | 100.0 | 47.23(0.58) | 2.59 | 48.27 | 494.11 |  | 46.84(0.66) | 0.01 | 48.27 | 494.11 |  | 49.31(1.44) | 3.42 | 48.28 | 405.26 | 0.13 |
| Cd | 7719 | 84.1 | 0.31(0.01) | 0.01 | 0.34 | 3.89 |  | 0.29(0.01) | 0.01 | 0.34 | 3.84 |  | 0.42(0.01) | 0.02 | 0.34 | 3.89 | ＜0.0001 |
| Ur | 7719 | 73.8 | 0.01 (0.01) | 0.00 | 0.01 | 3.55 |  | 0.01 (0.01) | 0.00 | 0.01 | 1.36 |  | 0.01 (0.01) | 0.00 | 0.01 | 0.28 | 0.56 |
| Tl | 7719 | 96.0 | 0.17(0.01) | 0.01 | 0.18 | 2.11 |  | 0.17(0.01) | 0.00 | 0.17 | 3.04 |  | 0.18 (0.09) | 0.02 | 0.17 | 9.69 | 0.52 |
| Pb | 7719 | 94.7 | 0.59(0.01) | 0.04 | 0.64 | 50.10 |  | 0.57(0.01) | 0.04 | 0.64 | 50.10 |  | 0.67(0.02) | 0.05 | 0.64 | 8.86 | ＜0.0001 |
| Tu | 7719 | 90.3 | 0.11(0.01) | 0.01 | 0.11 | 4.85 |  | 0.11(0.01) | 0.00 | 0.11 | 4.85 |  | 0.12(0.01) | 0.00 | 0.11 | 1.99 | 0.18 |
| Hg | 7719 | 93.1 | 0.62(0.02) | 0.01 | 0.60 | 57.10 |  | 0.62(0.02) | 0.00 | 0.60 | 35.88 |  | 0.63(0.02) | 0.00 | 0.60 | 57.10 | 0.86 |
| As | 7719 | 92.8 | 16.21(0.76) | 0.63 | 17.50 | 1441.17 |  | 15.87(0.76) | 0.00 | 17.50 | 1441.17 |  | 18.02(1.66) | 0.00 | 17.48 | 437.57 | 0.18 |

IQR, inter-quartile range; N, number.

**Table S2.** Associations of multiple urinary metals with psoriasis risk after adjustment for other metals in NHANES 2005-2016 cycles

| Metals | Q1 |  | Q2 | |  | Q3 | |  | Q4 | |  | Continuous | |
| --- | --- | --- | --- | --- | --- | --- | --- | --- | --- | --- | --- | --- | --- |
|  | OR (95% CI) |  | OR (95% CI) | *P* value |  | OR (95% CI) | *P* value |  | OR (95% CI) | *P* value |  | OR (95% CI) | *P* value |
| **Urine (μg/g creatinine)** | |  |  |  |  |  |  |  |  |  |  |  |  |
| Cd | Ref |  | **1.53(1.16,2.01)** | **0.004** |  | **1.82(1.39,2.39)** | **＜0.0001** |  | **2.01(1.51,2.67)** | **＜0.0001** |  | **1.28(1.15,1.43)** | **＜0.0001** |
| Co | Ref |  | 1.07(0.80,1.43) | 0.66 |  | 1.21(0.91,1.59) | 0.18 |  | **1.34(1.06,1.69)** | **0.02** |  | **1.11(1.00,1.24)** | **0.03** |
| Cs | Ref |  | 0.84(0.67,1.05) | 0.12 |  | 1.02(0.80,1.31) | 0.87 |  | 1.01(0.76,1.35) | 0.94 |  | 1.08(0.86,1.36) | 0.50 |
| Mo | Ref |  | 0.99(0.78,1.26) | 0.95 |  | 1.05(0.84,1.30) | 0.66 |  | 0.97(0.78,1.21) | 0.80 |  | 0.97(0.85,1.10) | 0.60 |
| Sb | Ref |  | 1.22(0.95,1.56) | 0.11 |  | **1.36(1.06,1.74)** | **0.02** |  | **1.36(1.04,1.79)** | **0.03** |  | **1.17(1.03,1.33)** | **0.01** |
| Tu | Ref |  | 0.81(0.63,1.05) | 0.11 |  | 0.78(0.61,1.01) | 0.06 |  | 1.03(0.80,1.33) | 0.79 |  | 1.04(0.92,1.18) | 0.49 |
| Ur | Ref |  | 1.13(0.88,1.45) | 0.33 |  | 1.10(0.82,1.47) | 0.52 |  | 1.22(0.95,1.58) | 0.12 |  | 1.04(0.93,1.15) | 0.50 |
| Tl | Ref |  | 0.99(0.80,1.22) | 0.90 |  | 0.79(0.59,1.05) | 0.10 |  | 0.92(0.71,1.19) | 0.50 |  | 0.90(0.74,1.10) | 0.31 |
| Pb | Ref |  | 1.07(0.85,1.36) | 0.53 |  | 1.17(0.92,1.51) | 0.20 |  | 1.24(0.95,1.61) | 0.10 |  | **1.14(1.02,1.28)** | **0.02** |
| Ba | Ref |  | 0.87(0.67,1.12) | 0.27 |  | **0.71(0.54,0.95)** | **0.02** |  | **0.70(0.53,0.92)** | **0.01** |  | **0.86(0.77,0.96)** | **0.01** |
| Hg | Ref |  | 0.98(0.77,1.23) | 0.84 |  | 1.12(0.89,1.41) | 0.31 |  | 0.87(0.66,1.13) | 0.28 |  | 0.96(0.87,1.05) | 0.32 |
| As |  |  | 0.99(0.81,1.21) | 0.92 |  | 1.01(0.83,1.23) | 0.92 |  | 0.88(0.70,1.11) | 0.27 |  | 0.97(0.88,1.07) | 0.50 |

Model was adjusted for 12 urinary metals, age, sex, race/ethnicity, education, Poverty Income Ratio, marital status, body mass index, smoke, drinking status, diabetes, stroke, hypertension, urine creatinine, and NHANES cycles. Continuous, Ln-transformed concentration of metal; CI: confidence interval; OR: odds ratio; Q, quartile; Ref, reference.

**Bold**: *p* < 0.05.

**Table S3.** The posteriori inclusion probability of single urinary metals in the NHANES 2005-2016 cycles

|  | PIP value | | | | |
| --- | --- | --- | --- | --- | --- |
| Metals | Total | 20 ≤ Age < 60 | Age ≥ 60 | Ever drink | Never drink |
| Urine (μg/g creatinine) |  |  |  |  |  |
| Cd | 0.99 | 0.99 | 0.77 | 0.99 | 0.78 |
| Co | 0.98 | 0.53 | 0.88 | 0.91 | 0.67 |
| Ba | 0.97 | 0.67 | 0.78 | 0.94 | 0.64 |
| Tl | 0.92 | 0.69 | 0.77 | 0.62 | 0.86 |
| Cs | 0.86 | 0.57 | 0.89 | 0.51 | 0.79 |
| Mo | 0.71 | 0.98 | 0.63 | 0.54 | 0.72 |
| Ur | 0.69 | 0.57 | 0.58 | 0.51 | 0.79 |
| Pb | 0.63 | 0.56 | 0.59 | 0.55 | 0.65 |
| Sb | 0.55 | 0.52 | 0.60 | 0.57 | 0.78 |
| Hg | 0.54 | 0.59 | 0.72 | 0.51 | 0.70 |
| Tu | 0.53 | 0.52 | 0.57 | 0.53 | 0.67 |
| As | 0.53 | 0.56 | 0.66 | 0.52 | 0.64 |

PIP, posteriori inclusion probability.


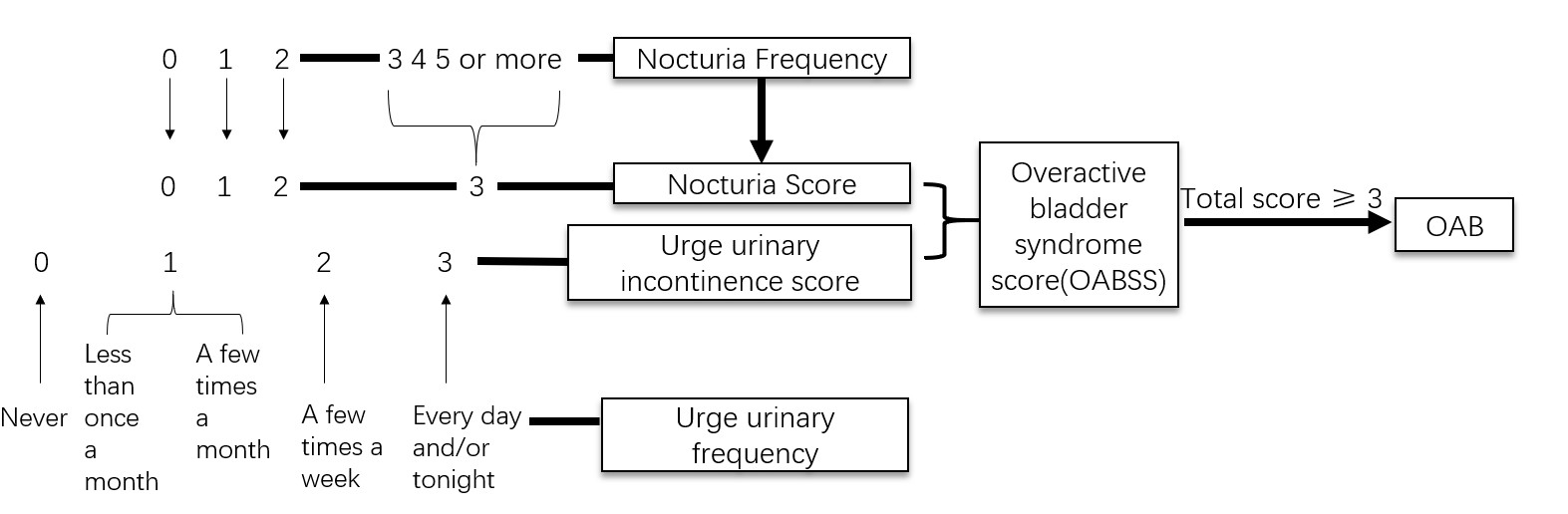


**Fig. S1** Flow diagram of the screening and enrollment of study participants.


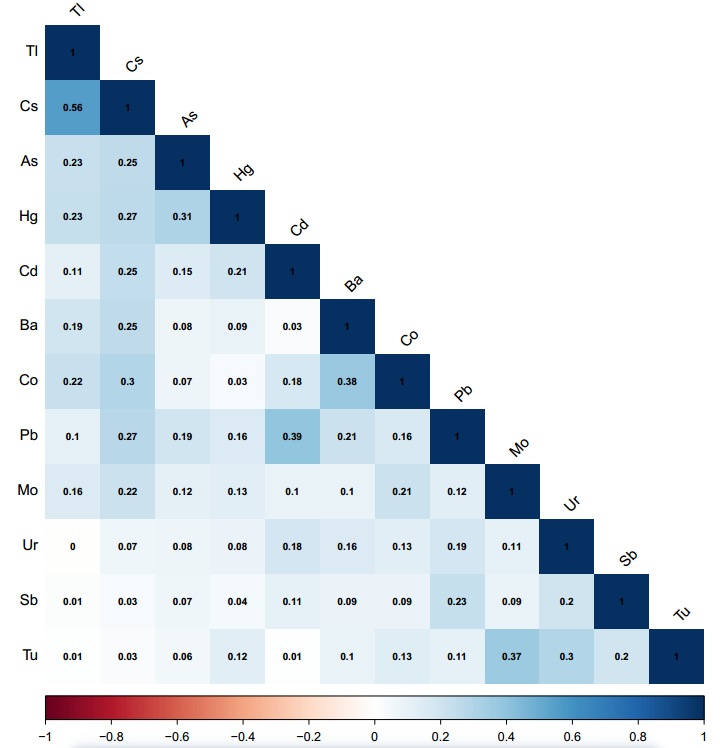


**Fig. S2** The Pearson correlation between metals after In-transformed.


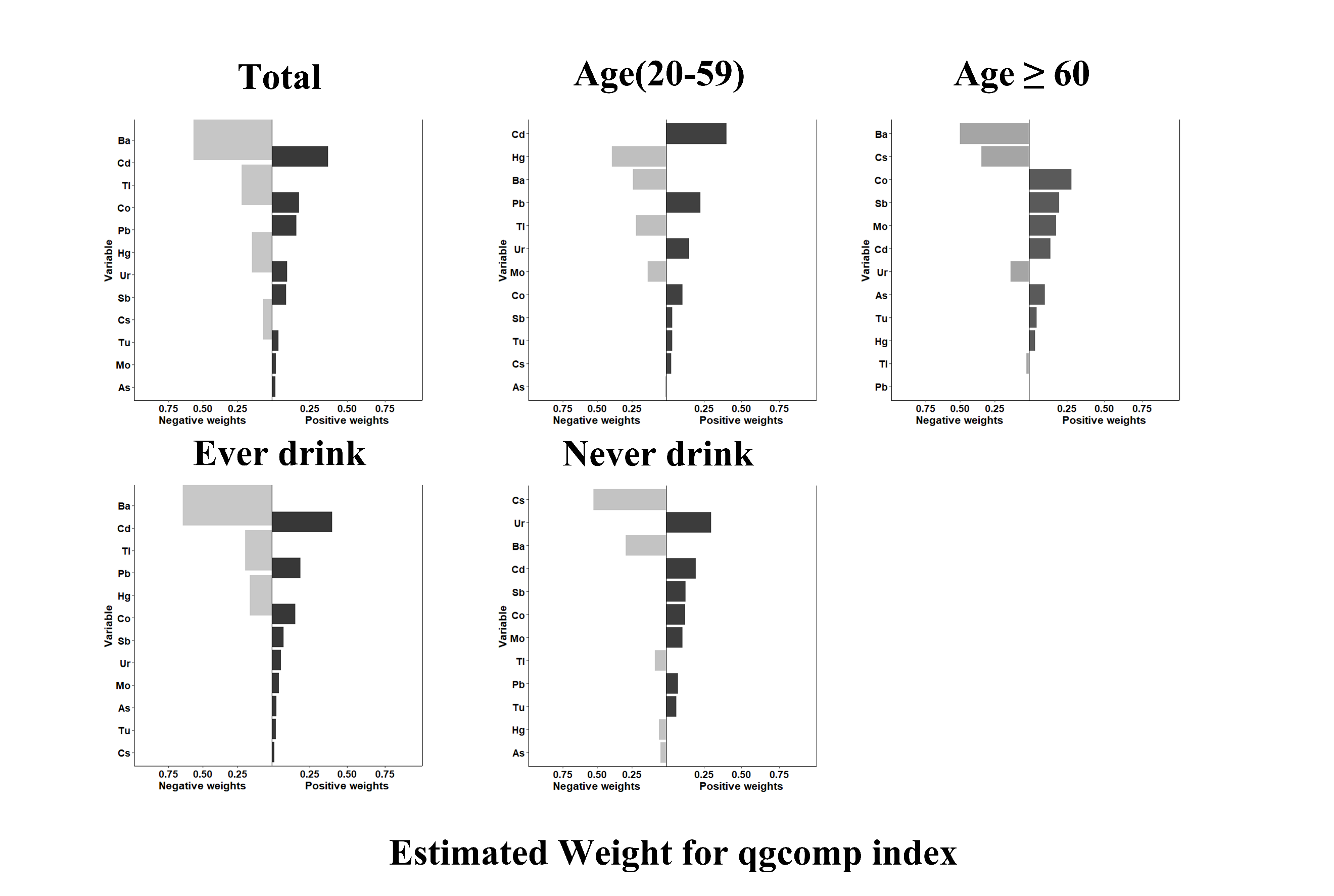


**Fig. S3** The positive and negative weights of metals for OAB by qgcomp models adjusted for age, sex, race/ethnicity, education, Poverty Income Ratio, marital status, body mass index, smoke, drinking status, diabetes, stroke, hypertension, urine creatinine, and NHANES cycles.


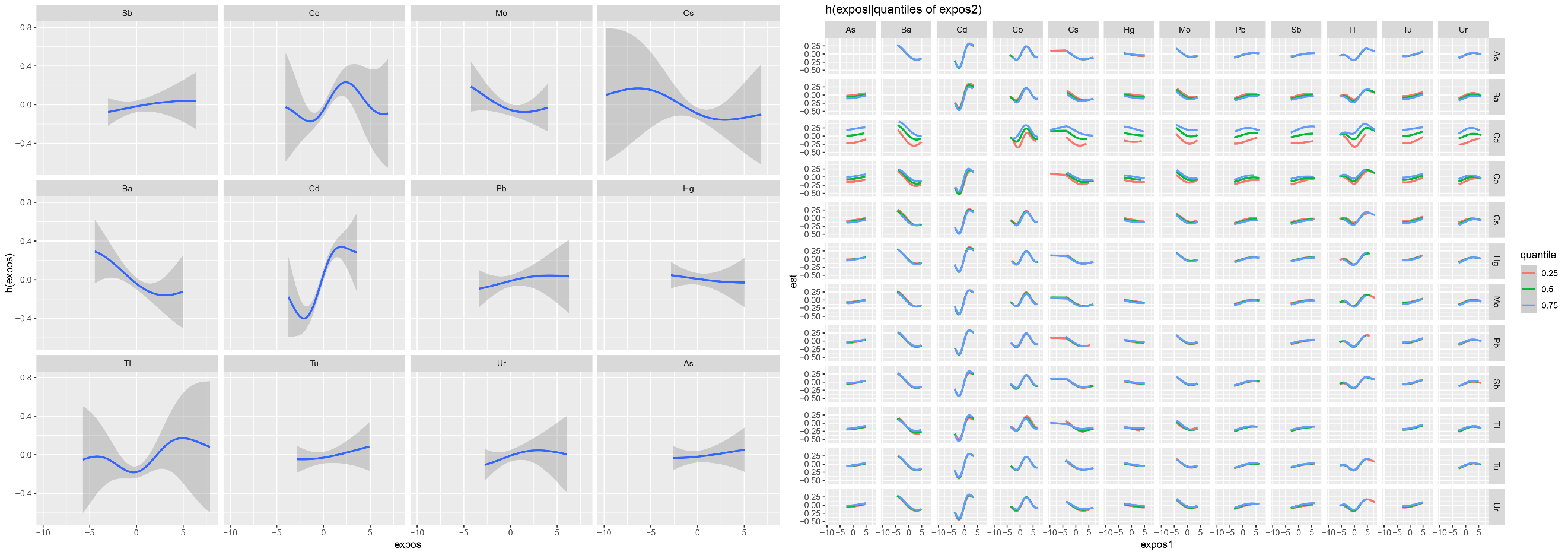


**Fig. S4** The interaction of urinary metals for psoriasis by BKMR models adjusted for age, sex, race/ethnicity, education, Poverty Income Ratio, marital status, body mass index, smoke, drinking status, diabetes, stroke, hypertension, urine creatinine, and NHANES cycles.
